# Systemic Gaps and Roadmap for Integrated Care for Post-tuberculosis Lung Disease in Ethiopia: A Narrative Review

**DOI:** 10.7759/cureus.109856

**Published:** 2026-05-29

**Authors:** Abraham T Ajema, Surafel N Firdawok, Tizita Shimelis, Ermit M Dashew, Kirubel T Hailu, Tariku Tesfaye, Ryan R Haddad

**Affiliations:** 1 Department of Medical/Clinical Services, AIDS Healthcare Foundation, Hawassa, ETH; 2 Department of Internal Medicine, College of Medicine and Health Sciences, Hawassa University, Hawassa, ETH; 3 Department of Radiology, Yirgalem Hospital Medical College, Yirgalem, ETH; 4 Department of Public Health, University College Cork, Cork, IRL; 5 Department of Clinical Research, California Institute of Behavioral Neurosciences & Psychology, Fairfield, USA

**Keywords:** chronic fibrosing interstitial lung diseases, chronic lung diseases, ethiopia, post-tb lung diseases, pulmonary function after tuberculosis

## Abstract

Post-tuberculosis lung disease (PTLD) is a major cause of chronic respiratory impairment globally, yet it remains a critically neglected complication in high-burden, low-resource settings. This narrative review synthesizes evidence on PTLD, with a specific focus on Ethiopia, to analyze the health system gaps and propose actionable solutions. We conducted a narrative review of published and grey literature on the clinical burden, socioeconomic impact, and health system challenges of PTLD, with a particular emphasis on Ethiopia. Evidence was selected for relevance to PTLD phenotypes, diagnosis, management, and health system integration. PTLD, encompassing conditions like bronchiectasis, chronic obstructive pulmonary disease (COPD), and pulmonary fibrosis, affects a substantial proportion of tuberculosis (TB) survivors, leading to chronic respiratory symptoms, lung function disability, and socioeconomic impact. In Ethiopia, despite its inclusion as a priority lung disease in the national strategic plan, critical gaps persist. These include a profound lack of clinical and patient awareness, severely limited diagnostic capacity (spirometry, imaging), absent treatment and rehabilitation pathways, a scarcity of local epidemiological and implementation research, and unaddressed policy system barriers that impact infectious and chronic disease care. Ethiopia's policy recognition of PTLD is a pivotal first step. An urgent, coordinated effort is required to translate this into functional, equitable care. Success depends on concurrent action by researchers to generate local evidence, public health institutes to integrate surveillance and develop guidelines, clinicians to pioneer service delivery models, and policymakers to mandate and fund integration. Bridging the PTLD care gap is essential to safeguard the long-term health of TB survivors and strengthen the health system’s capacity to manage chronic disease.

## Introduction and background

Post-tuberculosis lung disease (PTLD) represents one of the most under-recognized and under-addressed sources of disability in global health [1]. PTLD is defined as evidence of chronic respiratory abnormality, with or without symptoms, attributable at least in part to previous tuberculosis (TB) [2]. It is characterized by chronic respiratory impairment, including bronchiectasis, fibrosis, and obstructive airflow limitation [3]. Clinically, PTLD manifests with persistent dyspnea, exercise intolerance, fatigue, and a reduced ability to participate in work and social life [4].

The burden of persistent respiratory symptoms, functional lung impairment, and structural abnormalities on imaging following TB is high. A systematic review and meta-analysis of studies conducted in low- and middle-income countries (LMICs) found that the prevalence of abnormal lung function was 46.7%, persistent respiratory symptoms were present in 41.0%, and radiologic abnormalities were observed in 64.6% of patients [5]. There are also various evidence suggesting more than half of individuals who successfully complete TB treatment exhibit some degree of abnormal lung function, with a substantial proportion experiencing moderate to severe impairment [6-8]. Yet, in health systems strained by acute care priorities and limited diagnostic capacity, PTLD remains largely invisible, uncounted in disease registries, unaddressed in clinical guidelines, and untreated in communities. This neglect transforms a biomedical success story, TB cure, into a long-term personal and social crisis [1]. In Ethiopia, PTLD has recently gained policy recognition as a priority lung disease, but local evidence remains limited and care pathways are not yet well established.

The consequences of this oversight are profoundly inequitable. In settings where livelihoods depend on physical labor and social safety nets are weak, PTLD entrenches poverty, disproportionately affects vulnerable groups, and exacerbates gender and economic disparities. This review synthesizes growing evidence on the burden, clinical spectrum, and socioeconomic impact of PTLD, arguing that its management is not merely a clinical issue but also a pressing health system and equity imperative. Without deliberate integration of post-TB care into primary health and chronic disease platforms, the gains made in TB control risk being undermined by a legacy of preventable disability, demanding an urgent realignment of policy, funding, and practice in global health.

## Review

Methods

We conducted a narrative review to synthesize evidence on the clinical burden, socioeconomic impact, and health system challenges of PTLD, with a focus on Ethiopia and comparable high TB burden, resource-limited settings. We searched PubMed and Google Scholar and reviewed relevant grey literature up to February 10, 2026.

Search terms were combined using keywords and Boolean operators and included: PTLD, post-TB, TB survivors, lung function, spirometry, bronchiectasis, COPD, pulmonary fibrosis, pulmonary rehabilitation, chronic pulmonary aspergillosis, health systems, integration, Ethiopia, and sub-Saharan Africa. Reference lists of key articles were screened to identify additional relevant sources.

We included peer-reviewed studies and grey literature addressing PTLD definitions and phenotypes, epidemiology and burden among TB survivors, functional impairment and quality-of-life outcomes, socioeconomic consequences, diagnostic approaches in low-resource settings, management strategies, and service delivery and health-system integration. Evidence was selected based on relevance to the review aims. Recent syntheses, consensus statements, and guidance documents were prioritized. Ethiopian studies were included when available, and evidence from comparable settings was used where local data were limited. Studies unrelated to PTLD or without post-treatment relevance were excluded.

Findings were synthesized thematically and organized around clinical features, diagnostic and management pathways, and health system barriers and opportunities relevant to Ethiopia. Where evidence was limited or inconsistent, this was noted explicitly and causal inferences were avoided.

Because this was designed as a narrative review rather than a systematic review or meta-analysis, no formal risk-of-bias assessment, PRISMA flow diagram, or quantitative evidence pooling was performed. The purpose was to synthesize clinically and policy-relevant evidence and identify health-system gaps relevant to Ethiopia. No meta-analysis, meta-regression, or new statistical analysis was performed. Quantitative estimates cited in this review were extracted from included studies and prior systematic reviews and are presented descriptively to support the narrative synthesis.

Overview Post-TB Lung Disease 

Clinical Overview

Patients after completion of anti-TB therapy will be declared cured, microbiologically, but certain symptoms and/or lung functional impairments could arise as part of post-TB lung disease. These symptoms could be shortness of breath, cough, chest pain, fatigue, or hemoptysis [3]. Additionally, depending on the extent of lung damage, patients could be dependent on supplemental oxygen after successful completion of TB treatment [1].

In a Ugandan study, 70% of participants (140 individuals) rated their overall health as good to very good. However, 58% reported having at least one symptom after completing TB treatment. The most common symptom was cough, affecting 51% of participants, while the least frequently reported were night sweats (4%) and blood in sputum (2%). Only 16.5% of participants experienced more than one symptom [9].

TB survivors described negative experiences with lingering or recurring respiratory symptoms such as persistent cough, hemoptysis, or chest pain even after completing TB treatment. Their accounts were largely characterized by feelings of uncertainty, fear, and anxiety. Those who still had symptoms at the end of treatment expressed considerable doubt, questioning why they had not improved and whether the TB medications had been effective [10].

Patterns of PTLDs

The patterns of lung damage caused by TB are diverse and vary both between and within individuals. These patterns may occur with or without symptoms, and an individual patient may have multiple patterns [3].

Major post-pulmonary TB complications include traction bronchiectasis, COPD-like airflow obstruction, chronic pulmonary aspergillosis or intracavitary fungal ball, hemoptysis, extensive parenchymal destruction causing respiratory insufficiency, pulmonary hypertension or cor pulmonale, and pleural sequelae such as fibrothorax after pleural disease.

The effects of TB on the airways range from tracheal and bronchial stenosis, bronchiectasis, to small airways dysfunction and obliterative bronchiolitis. These factors lead to respiratory symptoms marked by chronic and irreversible airflow limitation, which is worsened by inhaling noxious particles and gases and is typically diagnosed as COPD. More than half of COPD cases worldwide are unrelated to tobacco smoking and are increasing in low-income countries. Common non-tobacco-related causes of COPD are recurrent bacterial infections and TB [11,12].

Most bronchiectasis is reported to be idiopathic; however, post-infectious etiologies dominate across Asia, especially secondary to TB. It is associated with chronic progressive and irreversible dilatation of the bronchi and is characterized by chronic infection and associated inflammation [13].

Approximately half of bronchiectasis cases were attributed to previous TB. In these post-TB cases, chest CT scans frequently revealed fibrocavitary changes, cylindrical and varicose patterns of bronchiectasis, as well as the tram-track sign. Patients with post-TB bronchiectasis had significantly reduced lung function compared to those with other bronchiectasis types [14].

Among patients who completed TB treatment followed at the chest clinic of Tikur Anbessa Specialized Hospital (TASH), 29% had post-TB bronchiectasis [15].

Although most cavities heal and close after anti-TB chemotherapy, some persist beyond treatment. Incomplete healing might leave the cavity’s inner space exposed to the primary airway system, raising the susceptibility to secondary colonization by pathogens such as *Aspergillus fumigatus* [12,16,17]. Furthermore, PTLD could also present with various levels of damage to the respiratory system, such as lung parenchymal destruction, fibrotic change with associated lung volume loss, chronic pleural disease, and pulmonary hypertension [11].

Post-TB participants had a greater risk of obstructive pulmonary impairment and respiratory symptoms related to chronic pulmonary obstruction [18]. A study conducted in India on post-TB patients found that among symptomatic individuals assessed by spirometry, the obstructive pattern was the most common, occurring in 42% of cases. Among asymptomatic post-TB patients, spirometry revealed an obstructive pattern in 32%, a mixed pattern in 14%, and normal spirometry results in 46% of cases [19].

Gaps in Care and Policy in Ethiopian Context

Awareness gap: There is a common lack of awareness among healthcare providers managing TB as well as patients undergoing TB treatment regarding the chronic sequelae of TB. In typical TB-focused investigations and routine care, when patients return with chronic respiratory issues, the initial evaluation primarily aims to exclude recurrent TB and drug-resistant disease. Little attention is given to the broader spectrum of possible cardiorespiratory conditions [10]. There is no widespread understanding that chronic cough, breathlessness, and fatigue after TB treatment are not normal but could be signs of a new chronic condition requiring further evaluation and treatment. In recently published case from Zambia where there is a high TB burden and similar to Ethiopia with isolated active TB care, the lack of awareness of the burden of PTLD among healthcare providers resulted in late diagnosis of PTLD and misdiagnosis as TB relapse, leading to inappropriate anti-TB initiation [20]. 

A critical yet often overlooked gap in Ethiopia's response to PTLD lies in the pre-service training of healthcare providers. Despite TB being one of the country's most significant infectious diseases, the long-term sequelae of TB remain inadequately addressed in the curricula of medical schools and allied health professional training institutions. PTLD is rarely featured in seminars, lectures, or departmental grand rounds, leaving graduate providers with limited exposure to and understanding of post-TB complications. As a result, primary healthcare providers often enter the workforce unprepared to recognize, assess, or appropriately manage patients with PTLD, perpetuating the cycle of under-diagnosis and neglect. This educational gap undermines efforts to build a health workforce capable of delivering comprehensive, long-term care to the growing population of TB survivors.

In Ethiopia’s national TB program, a major milestone in awareness creation of PTLD has come from the recent Tuberculosis, Leprosy, and other Lung Diseases National Strategic Plan for July 2023-June 2030, which listed PTLD as a priority lung disease that could benefit from programmatic interventions. Most of the interventions outlined in the plan focus on increasing epidemiological knowledge, setting up effective and efficient service delivery models, and raising awareness of policymakers, health workers, and affected and at-risk populations. While this is a landmark for acknowledging PTLD, awareness must now translate into budgeted, actionable programs within the existing health system, not just a listed priority [21].

Diagnostic and treatment gaps: Spirometry remains the primary method for evaluating lung function impairment following TB treatment, but it is rarely available outside large tertiary hospitals. In Africa, access to spirometry is restricted due to several factors: inadequate training opportunities and a shortage of skilled technicians, limited availability of equipment, consumables, and technical support, as well as a lack of both personnel and financial resources [22]. 

Imaging plays a key role in evaluating PTLD. Chest X-ray (CXR) and computed tomography (CT) of the lungs are the most commonly used techniques for assessing TB and its long-term effects. A high proportion of patients still show residual structural lung abnormalities after completing PTB treatment, underscoring the value of CT imaging in helping us better understand these changes. However, access to such advanced imaging may be limited. The variability in predominant radiologic findings between CXR and CT, limited comparative studies between lung pathology, symptoms, and spirometer tests make the diagnosis even more challenging [23].

Furthermore, there is no practical and operationalized national clinical guidance being implemented on how to diagnose PTLD and no structured system for linking patients completing TB treatment to respiratory follow-up at ground level to execute the limited guidance provided in a national guidance.

Additionally, there are no standard protocols for managing PTLD [17,24]. This includes pharmacotherapy and non-pharmacological interventions as well as management of complications like chronic pulmonary aspergillosis or cor pulmonale. Furthermore, pulmonary rehabilitation, which is a cornerstone of managing chronic lung disease, is non-existent as a structured service in the public health system [1,25,26].

Research gaps: The true burden, phenotypes (obstructive, restrictive, bronchiectasis, destroyed lung), and socio-economic impact of PTLD in Ethiopia are poorly quantified. Without these data, advocating for resources is difficult. Although facility-based and regional Ethiopian studies are emerging, Ethiopia lacks nationally representative PTLD data, including prevalence, clinical phenotypes, severity, quality-of-life impact, economic consequences, and care-seeking pathways.

There is also a lack of understanding regarding how PTLD progresses over time following TB. Evidence from prospective studies is needed to clarify the trajectory of imaging-defined post-TB lung disease [27]. A similar limitation exists in understanding how lung function changes after pulmonary tuberculosis (PTB), as the available evidence is derived primarily from cross-sectional studies rather than longitudinal data. Research on mental health (anxiety, depression), stigma, and the impact on livelihoods post-TB are severely lacking [28,29].

Policy and systems gap: The inclusion of PTLD in the national Strategic Plan is step one. The next steps, developing technical guidelines, training curricula, budget lines, and M&E frameworks are yet to be fully realized. There is no dedicated funding for PTLD care. Services like spirometry, imaging, and rehabilitation could be challenging for post-TB patients.

Existing health information systems (like DHIS2) do not capture PTLD data. There is no code or register to track the diagnosis, management, or outcomes of these patients, making them invisible to the system. Effective management requires bridging the TB program (vertical, infectious disease focus) with the NCD or chronic care program (horizontal, long-term management focus). This structural gap is a major barrier.

Recommendations

**Figure 1:**
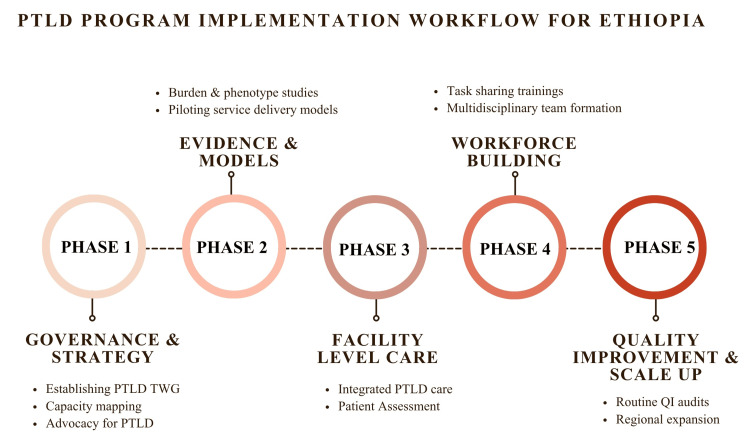
Phased implementation roadmap for integrated PTLD care in Ethiopia. The figure illustrates a five-phase roadmap for implementing PTLD care in Ethiopia, including governance and strategy, evidence and models, facility-level care, workforce building, and quality improvement with scale-up. Image Credit: Original figure created by Abraham Teka Ajema using Canva. PTLD: post-tuberculosis lung disease; TWG: Technical Working Group; QI: quality improvement.

Ethiopia has taken an important first step by recognizing PTLD as a priority lung disease in the national strategic plan. The next step is to translate this recognition into practical care through coordinated action in research, policy, clinical service delivery, workforce development, and monitoring. The proposed national PTLD program implementation workflow for Ethiopia is summarized in Figure 1.

For researchers and academia: Researchers should generate locally relevant evidence on the burden, phenotypes, severity, and socioeconomic impact of PTLD in Ethiopia. Multicenter studies are needed to estimate prevalence, describe common clinical patterns, and assess outcomes such as quality of life, stigma, mental health, job loss, and household economic burden.

Implementation research should evaluate feasible models of PTLD care within existing TB, respiratory, and chronic disease services. Priority areas include simplified diagnostic pathways, task-shifted pulmonary rehabilitation, referral systems, cost-effectiveness, and patient-centered outcomes.

Academic institutions should also strengthen pre-service and in-service training on PTLD. Medical schools and allied health programs should include PTLD in teaching sessions, clinical case discussions, and continuing professional development activities so that future providers can recognize, manage, and refer patients with post-TB complications.

For public health institutes and policymakers: Public health institutes and policymakers should move from policy recognition to implementation. A national PTLD task team within the existing TB Technical Working Group could coordinate guideline development, surveillance, training, financing, and service integration.

Ethiopia should develop practical clinical guidance for PTLD screening, diagnosis, referral, and management. Core PTLD indicators should also be integrated into routine health information systems, including the proportion of TB treatment completers screened for respiratory symptoms and the proportion linked to further care.

Policymakers should map diagnostic and rehabilitation capacity, including spirometry, imaging, oxygen availability, essential medicines, trained personnel, and pulmonary rehabilitation services. This mapping can guide phased investment, beginning with referral hospitals and later expanding to lower levels of care. Dedicated financing is needed to support implementation. Donor and partner support should be aligned with national priorities and should strengthen sustainable services rather than create parallel programs.

For clinicians and healthcare facilities: Clinicians and healthcare facilities should begin integrating PTLD care into routine post-TB follow-up. Facilities can start with symptom screening at treatment completion and referral of patients with persistent cough, dyspnea, hemoptysis, chest pain, or functional limitation.

Referral hospitals can pilot structured PTLD services within existing chest, TB, or chronic disease clinics. These services should provide phenotype-oriented assessment, spirometry where available, imaging review, treatment of complications, pulmonary rehabilitation referral, and long-term follow-up.

Task sharing should be used to make services feasible. Nurses and health officers can be trained to perform symptom screening, basic spirometry, patient education, and referral. Radiology teams can support standardized reporting of post-TB imaging findings. Facilities should document patient outcomes and implementation lessons. These data can support quality improvement, inform national guidelines, and guide future scale-up of PTLD services across Ethiopia.

## Conclusions

PTLD represents a silent yet pervasive burden of chronic disability arising from the very success of Ethiopia's TB control efforts. While the inclusion of PTLD in the national strategic plan offers a crucial policy mandate, translating this recognition into meaningful action requires urgently addressing the profound gaps in awareness, diagnostic capacity, treatment access, research evidence, and health system integration that currently impede care.

Bridging these gaps demands a phased and collaborative approach that harnesses the strengths of all stakeholders from researchers generating locally relevant evidence to frontline clinicians delivering patient-centered care, and from policymakers shaping guidelines to educators reforming pre-service curricula. Through such coordinated effort, Ethiopia can move beyond policy rhetoric to deliver tangible improvements in the health and well-being of TB survivors.
